# *Phormidium autumnale* Growth and Anatoxin-a Production under Iron and Copper Stress

**DOI:** 10.3390/toxins5122504

**Published:** 2013-12-16

**Authors:** Francine M. J. Harland, Susanna A. Wood, Elena Moltchanova, Wendy M. Williamson, Sally Gaw

**Affiliations:** 1 Department of Chemistry, University of Canterbury, Private Bag 4800, Christchurch 8140, New Zealand; E-Mail: francine.smith@canterbury.ac.nz; 2 Cawthron Institute, Private Bag 2, Nelson 7042, New Zealand; E-Mail: susie.wood@cawthron.org.nz; 3 Department of Biological Sciences, University of Waikato, Private Bag 3105, Hamilton 3240, New Zealand; 4 Department of Mathematics and Statistics, University of Canterbury, Private Bag 4800, Christchurch 8140, New Zealand; E-Mail: elena.moltchanova@canterbury.ac.nz; 5 Institute of Environmental Science and Research, PO Box 29-181, Fendalton Christchurch 8540, New Zealand; E-Mail: wendy.williamson@esr.cri.nz; 6 Biomolecular Interaction Centre, School of Biological Sciences, University of Canterbury, Private Bag 4800, Christchurch 8140, New Zealand

**Keywords:** anatoxin-a, benthic cyanobacteria, cyanotoxin production, metal stressors, *Phormidium autumnale*

## Abstract

Studies on planktonic cyanobacteria have shown variability in cyanotoxin production, in response to changes in growth phase and environmental factors. Few studies have investigated cyanotoxin regulation in benthic mat-forming species, despite increasing reports on poisoning events caused by ingestion of these organisms. In this study, a method was developed to investigate changes in cyanotoxin quota in liquid cultures of benthic mat-forming cyanobacteria. Iron and copper are important in cellular processes and are well known to affect growth and selected metabolite production in cyanobacteria and algae. The effect of iron (40–4000 μg L*^−^*^1^) and copper (2.5–250 μg L*^−^*^1^) on growth and anatoxin-a quota in *Phormidium autumnale* was investigated in batch culture. These concentrations were chosen to span those found in freshwater, as well as those previously reported to be toxic to cyanobacteria. Anatoxin-a concentrations varied throughout the growth curve, with a maximum quota of between 0.49 and 0.55 pg cell*^−^*^1^ measured within the first two weeks of growth. Growth rates were significantly affected by copper and iron concentrations (*P* < 0.0001); however, no statistically significant difference between anatoxin-a quota maxima was observed. When the iron concentrations were 800 and 4000 μg L*^−^*^1^, the *P.* *autumnale* cultures did not firmly attach to the substratum. At 250 μg L*^−^*^1^ copper or either 40 or 4000 μg L*^−^*^1^ iron, growth was suppressed.

## 1. Introduction

The number of benthic cyanobacterial species known to synthesize cyanotoxins has increased dramatically over the past decade [1,2,3,4]. Although benthic species have received less attention than their planktonic counterparts, they are a significant problem in some parts of the world. For example, animal fatalities have been associated with benthic neurotoxin and hepatotoxin producing cyanobacteria in Africa, Australasia, Europe, and North America [4,5,6]. In New Zealand, the mat-forming and anatoxin-a-producing species *Phormidium autumnale* [Agardh] Trevisan ex Gomont has caused numerous dog poisonings during the last decade [7,8]. Proliferations of *P. autumnale* also pose a significant health risk to humans where rivers are used extensively for recreational activities and drinking water supplies [7,9].

The biological function of cyanotoxins is highly debated and hypotheses include defence mechanisms, nutrient or other metabolic storage reserves, antifouling agents, or to reduce abiotic stress [10,11]. It has also been suggested that cyanotoxins are produced in response to environmental stressors [11]. Environmental factors may have synergistic, antagonistic, or additive effects on cyanobacterial growth and metabolite production, including cyanotoxin biosynthesis [6,12,13]. Understanding the influence of environmental stressors on cyanotoxin production, as well as cyanobacterial growth, is essential for effective water management [14,15,16]. Laboratory studies provide an opportunity to explore how individual chemical and physical parameters impact cyanotoxin production. 

Metals are potential stressors for cyanobacterial and eukaryotic algae. These organisms readily accumulate metals from their environment [17,18,19,20]. Metals can enter aquatic habitats through leaching and run-off from mining, agriculture and industry as well as from natural geological sources [19]. Metal stressors have been reported to affect cyanobacterial growth [21] and alter the metabolites produced [22]. Exposure to non-essential metals or elevated concentrations of essential metals can be highly toxic to cells [17,21]. Similarly, limited availability of essential metals can also stress organisms. For example, Maldonado *et al.* [23] demonstrated that neurotoxic domoic acid production in the diatom *Pseudo-nitzschia* spp. can be activated by an increase in copper or reduction in iron availability.

Studies investigating the effects of metal stressors on cyanotoxin production are limited. Research on microcystin production by *Microcystis* spp. has provided variable results [6,24]. Iron deprivation, the most commonly studied metal stressor, in *Microcystis* spp. has been reported to both increase and decrease microcystin production [24,25,26,27,28]. In *Cylindrospermopsis raciborskii* [Woloszynska] Seenayya et Subba Raju, iron limitation reduces the growth rate indirectly effecting cylindrospermopsin production [29].

The present study investigates the effects of copper and iron on growth and anatoxin-a production by *P. autumnale*. Batch cultures were set-up and harvested at ten sampling points over a seven week period. Cultures were incubated until harvest date and replicate cultures were collected to determine cell and anatoxin-a concentrations, and to assess concentration of metals present in the culture media. Iron and copper were selected for the experiment as both are essential trace metals for physiological function in cyanobacteria. However, excess or limited availability of these metals can cause physiological stress and cellular concentrations must be tightly regulated [17,21]. Increased understanding of the effect of these essential metals on *P. autumnale* growth and anatoxin-a regulation will assist in predicting periods of highest health risk during benthic mat proliferation.

## 2. Results and Discussion

### 2.1. Media Concentrations

**Figure 1:**
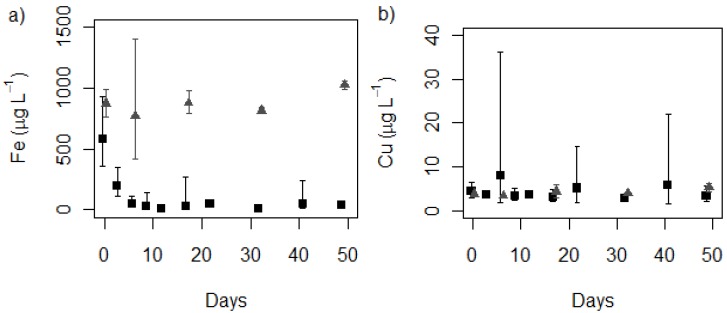
Mean metal concentrations in MLA with 95% confidence intervals measured in *Phormidium autumnale* (CYN52) growth experiments: (**a**) Iron 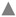 control, ■ treatment (MLA_2×Fe_); (**b**) Copper 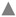 control, ■ treatment (MLA_2×Fe_). (*n* = 3 for cultures, *n* = 2+ for controls).

Concentrations of dissolved iron, copper and nutrients (nitrate, nitrite, and phosphorus) in MLA from all samples were compared with controls, which contained no *P.* *autumnale*. Nutrient concentrations remained relatively constant throughout the experiment in both control and culture medium (data not shown). In each treatment, iron concentrations decreased rapidly but remained constant in the controls (Figure 1a, Supporting Information Figure S1). 

The control data confirm that iron was not removed from the culture medium via sorption to the container walls, but that the decreases were due to biological factors. Strain CYN52 was unialgal but non-axenic, making it likely that this decrease is attributed to metal interactions with the *P. autumnale* strain and associated bacteria and viruses. Even low quantities of bacteria can accelerate iron removal from liquid cultures [30].

The iron interactions with strain CYN52 are also likely to be due to a combination of adsorption onto the outer cell wall and absorption into cells. Initial microbial metal uptake mechanisms usually consist of rapid adsorption to the cell wall, followed by a longer metabolism dependent phase transporting the metal into the cell [18,19,31]. The largest decrease in iron was observed under nominal 800 μg L^−1^ Fe conditions (MLA_2×Fe_). At this concentration, the control and culture were initially measured at 876 ± 119 and 617 ± 276 μg L^−1^ Fe respectively. The slightly lower iron concentrations recorded in cultures on Day 0 may be explained by rapid adsorption of iron onto the cell surface occurring between inoculation and removal of a subsample for ICP-MS analysis (≤2 h). Singh *et al.* [32] observed a similar phenomenon in *Microcystis* cultures, where rapid uptake was observed within 20 min of exposure to iron. This initial uptake is assumed to be caused by passive surface adsorption. An increase in copper concentration did not appear to change the biosorption of iron in CYN52. This finding was consistent with the results of Singh *et al.* [32] who reported that copper did not affect iron biosorption.

Changes in copper concentrations were not observed under any of the treatments compared with the controls (Figure 1b, Supporting Information Figure S2). Singh *et al.* [32] observed iron had an antagonist effect on the biosorption of copper. However, this difference was observed for very high copper and iron concentrations (20000–50000 µg L^‑1^ range), which are not likely to be encountered in the natural environment. Metal concentrations tested in this study were well below these extremes. 

### 2.2. Growth Profile

**Figure 2:**
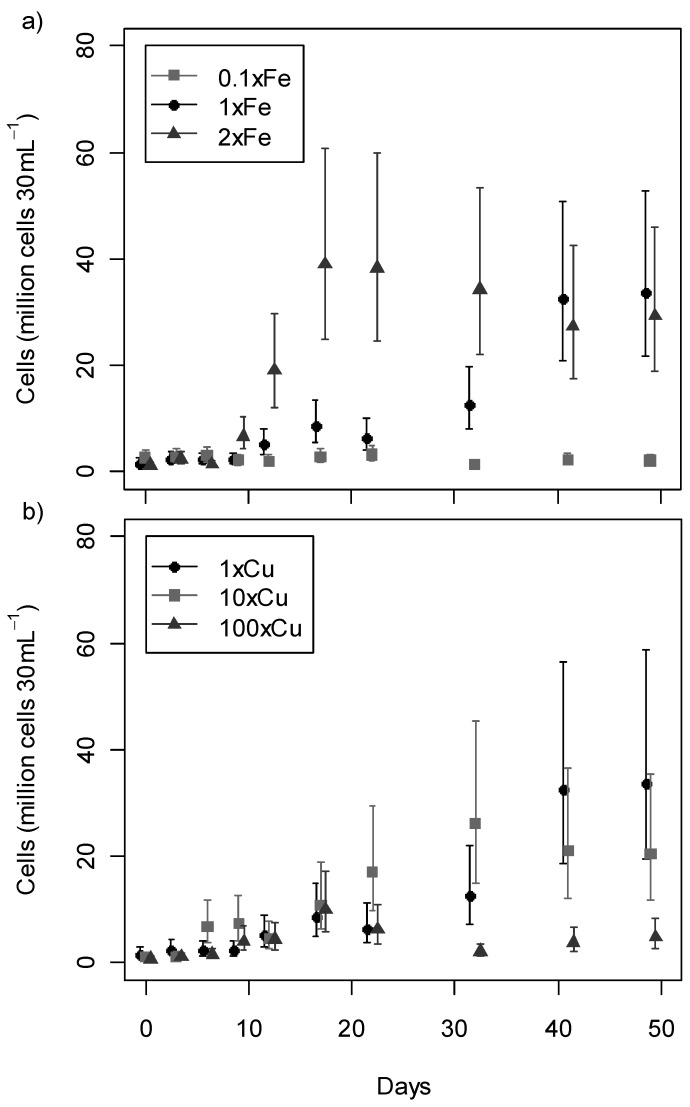
Growth profile for *Phormidium autumnale* (CYN52) under (**a**) iron (Fe) and (**b**) copper (Cu) regimes over 49 days. Growth is recorded as mean number of cells per 30 mL culture with 95% confidence intervals (*n* = 3).

The growth profile (Figure 2) of most treatments followed the typical stages of lag, exponential, and stationary phase. During the stationary phase the mat usually started to detach from the container wall. This trend was not observed under the lowest (MLA_0.1×Fe_, 40 μg L ^‑1^) and highest (MLA_10×Fe_, 4000 μg L^‑1^) iron concentrations tested, in which strain CYN52 struggled to survive. The majority of cultures in MLA_10×Fe_ did not survive past 14 days at which point the experiment was terminated for this treatment. Data from MLA_10×Fe_ were excluded from subsequent growth and anatoxin-a analyses.

It was not appropriate to use a single model to describe the growth curves for all treatments due to the large variation in growth profile observed between the treatments. The growth data were not well-described by logarithmic or sigmoidal curves. This observation was supported by Akaike Information Criteria (Supporting Information Table S1) where the models using days as categorical rather than a continuous variable provided the best fit. To aid the comparison of growth between treatments and through time, 95% confidence intervals were obtained for the expected value of cell counts (Figure 2) for each day-and-treatment combination.

Both iron and copper had a significant effect on the growth of CYN52 (Figure 2). Two-way ANOVA was used to test the factors metal treatment, day, and the interaction term (for non-additive effects). All these factors were significant (*P* < 0.0001) in describing the effect on growth. High copper concentrations (MLA_100×Cu_, 250 µg L^‑1^) and both low (MLA_0.1×Fe_, 40 µg L^‑1^) and high (MLA_10×Fe_, 4000 μg L^‑1^) iron concentrations reduced or inhibited growth. At high iron concentrations (MLA_2×Fe_ and MLA_10×Fe_) cultures did not attach firmly to container walls as observed in all other treatments. The concentration of iron in MLA_2×Fe_ (800 µg L^‑1^ Fe) has been detected in New Zealand streams [33]. Similar and higher concentrations can also arise from contamination through mining and industrial waste [34,35]. In these streams high iron concentrations could inhibit benthic proliferations of *P.* *autumnale* by preventing their attachment to the substratum.

Iron is an essential element that is involved in high energy pathways such as photosynthesis. Iron needs to be tightly regulated as many of these pathways also result in the formation of harmful free-radicals [17,21]. Strain CYN52 did not grow well under a very high (MLA_10×Fe_, 4000 µg L^‑1^ Fe) iron concentration. A reduction in growth attributed to oxidative stress induced by high iron concentrations has also been observed in cultures of green algae *Chlorella vulgaris* [36]. A detrimental effect on growth in high iron (5600 µg L^‑1^) conditions was also observed by Lukač and Aegerter [37]. Their study noted that cells adapted to iron deplete medium in *M. aeruginosa* cultures decayed when reintroduced to higher iron (1600 µg L^‑1^ Fe BG11) culture medium [37].

The reduction in growth observed in strain CYN52 under low iron conditions may also be explained by oxidative stress [38,39]. Gene expression induced under oxidative stress has been observed in *Anabaena* sp. under iron deficient conditions [40]. As oxidative stress was not observed in iron starvation of heterotrophic bacteria, Latifi *et al.* [38] proposed that only photosynthetic bacteria are susceptible to oxidative stress via iron limitation. Under a low Fe regime (MLA_0.1×Fe_, 40 µg L^‑1^ Fe) trichomes distributed across the container walls but cell concentrations did not increase notably and an exponential growth phase was not observed (Figure 2a). 

High copper concentrations were expected to have a detrimental effect on growth. Copper sulfate used as an algicide applied at concentrations between 10 and 1000 μg L^‑1^ is sufficient to eradicate most algae [41]. Although copper concentrations of 250 μg L^‑1^ (MLA_100×Cu_) had a significant negative effect on growth (Figure 2b), not all *P. autumnale* cells were killed. Cells initially grew under high copper concentrations, reaching a maximum of about 10 million cells at Day 17. *Phormidium autumnale* exposed to high copper concentration in the environment may acclimatise and become resistant to copper stress as has been previously shown in *Microcystis* cultures [42].

### 2.3. Anatoxin-a Production

The effect of stressors on anatoxin-a production is not well studied and research to date has focused only on planktonic species. These studies have demonstrated that anatoxin-a production can vary throughout the growth cycle. Profiles describing fluctuation in anatoxin-a production vary between species [43,44] and under different environmental conditions [44,45]. Anatoxin-a production is strain-dependent and strongly influenced by light, temperature and nutrient concentrations [46]. The effect of metal stressors on anatoxin-a production has not been investigated previously. 

**Figure 3:**
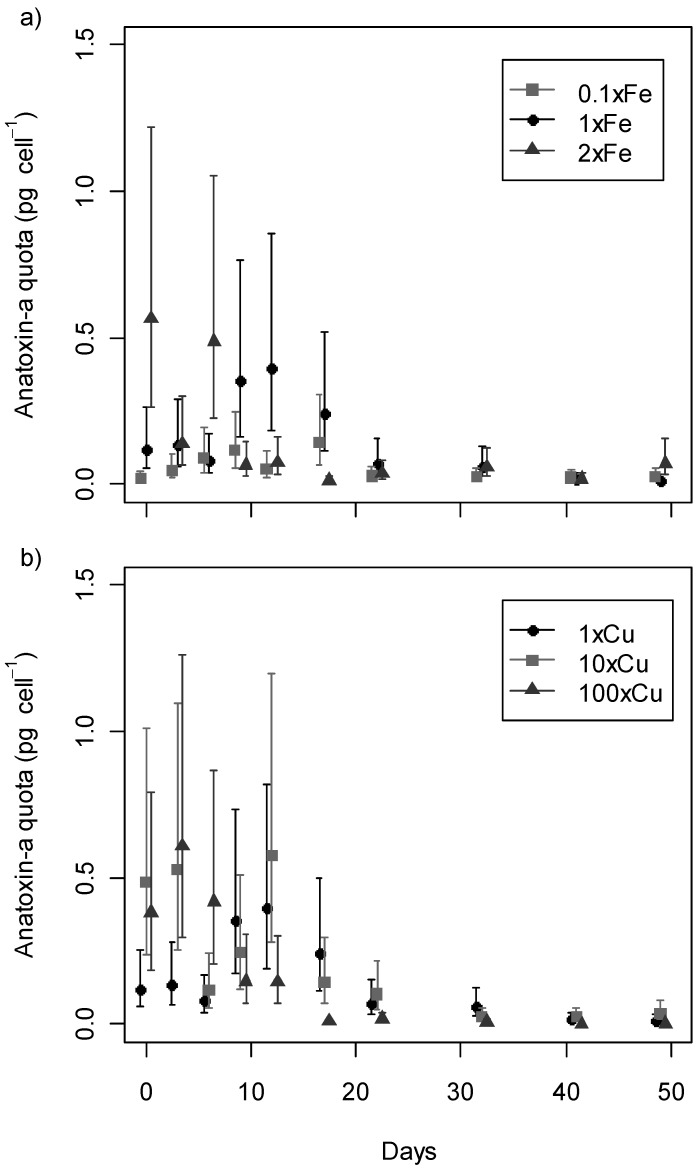
Intracellular anatoxin-a in *Phormidium autumnale* (CYN52) under (**a**) iron (Fe) and (**b**) copper (Cu) regimes. Anatoxin-a quota are recorded as mean concentrations with 95% confidence intervals (*n* = 3).

Anatoxin-a production was not completely inhibited by any concentration of the metal stressors tested. Anatoxin-a (<0.01–0.55 pg cell^−1^) was detected at all sampling points (Figure 3). While dihydro-anatoxin-a was occasionally detected in low concentrations (1–151 fg cell^−1^), homoanatoxin-a or dihydrohomoanatoxin-a were not detected.

The anatoxin-a concentration was not proportional to the number of cells. Model comparison demonstrated that the increase in anatoxin-a quota with time was non-linear (Supporting Information Table S2). Two-way ANOVA indicated that the effects of metal concentration, day, and their interaction on anatoxin-a quota were all significant (*P* < 0.0001). Toxin quota measures toxin concentration normalised to cell concentrations, therefore the significant changes in quota could be due to indirect influence on growth and not actual variation in anatoxin-a production. The quota maxima values were not statistically different between treatments in the cases where metal stressors did not inhibit growth.

In all treatments, the maximum intracellular anatoxin-a quota occurred within the first 20 days, usually during the late lag-phase and early exponential growth phase (Figure 3). Selwood *et al.* [47] observed a similar trend with the maximum anatoxin-a quota of *Aphanizomenon issatschenkoi* during the early exponential growth phase. In contrast, Gupta *et al.* [45] found that the anatoxin-a quota maximum for *Anabaena flos-aquae* occurred in the late exponential or early stationary phase. However, Gupta *et al.* [45] measured anatoxin-a using bioassays and not an anatoxin-a specific technique such as mass-spectrometry used in the current study. Microcystin quota within *Microcystis* has been reported to be directly positively correlated with growth rate in some studies [48,49,50,51].

The growth phase at which anatoxin-a production maxima occurs, may vary under different culturing conditions and between different species [44]. In this study, the maximum anatoxin-a quota usually coincided with spread of trichomes over all the available surface area of the container. All anatoxin-a quota maxima were statistically significantly higher than the quota recorded for the same treatment in the late sampling points of the experiment from Day 22–49. After Day 20, cell quotas were less than 0.1 pg cell^−1^, and this level was maintained under all media conditions for the remainder of the experiment. Anatoxin-a maxima were approximately 0.53 pg cell^−1^ (Figure 3) for all media except MLA_0.1×Fe_, which displayed little change in anatoxin-a quota and cell concentration (Figure 2). In contrast, growth was reduced in treatment MLA_100×Cu_, but not as significantly as MLA_0.1×Fe_, and a toxin quota of about 0.61 pg cell^−1^ was recorded.

Rapala *et al.* [44] reported anatoxin-a production maxima occurred in early, mid, or late exponential phase, which varied between *Anabaena* spp. and *Aphanizomenon flos-aquae* grown under various light, nutrient, and temperature conditions. However, in their study, Rapala *et al.* [44] reported anatoxin-a normalised to dry weight biomass rather than cell concentrations making it difficult to compare with our study. Correlation of cyanotoxin concentrations to different parameters, including dry weight, chlorophyll-a content, and protein content, makes comparison between studies difficult because these parameters may not be linearly correlated with cell counts. Negri *et al.* [52], illustrated this difficulty in *Anabaena circinalis* by observing significant changes in saxitoxin production correlated to biomass, but not when calculated as saxitoxin per cell (toxin quota). Issues can arise when using mass to normalise toxin production throughout the growth cycle. These issues include mass variability due to the presence of bacteria [53], and changes in carbohydrate storage as the culture ages [54]. Carbohydrate variability can affect the mass of the cell without necessarily affecting cyanotoxin production [54].

Significant differences in anatoxin-a production among different treatments and days were visualised by plotting 95% confidence intervals for each anatoxin-a data point (Figure 3). The anatoxin-a quota maxima occurred in the lag-early exponential growth phase. These maxima were noticeably different, occurring between Day 0 and 17. However the anatoxin-a quota values recorded for each maximum did not change significantly between treatments, except when growth was inhibited. Therefore, any changes in anatoxin-a quota are probably due to an indirect effect from growth not toxin production.

Anatoxin-a quota increased to a maximum value during the early exponential growth phase, and decreased to ≤0.1 pg cell^−1^ for the stationary phase. The exception was MLA_0.1×Fe_ where there was no observable growth throughout the experiment. In the MLA_0.1×Fe_ treatment, the maximum anatoxin-a quota recorded only reached 0.15 pg cell^−1^. This value was recorded on Day 17, and is within the 95% confidence interval of the maxima anatoxin-a quota recorded at the iron concentrations MLA_1×Fe_ and MLA_2×Fe_. Research on other cyanotoxins has shown varying effects under iron deficiency. For example, Alexova *et al.* [55] detected highest microcystin quota in *M. aeruginosa* for iron deplete conditions. In this case, exponential growth of *M. aeruginosa* coincided with the maximum microcystin quota under all iron treatments. However, in the present study no exponential growth phase was observed for the low iron treatment MLA_0.1×Fe_ (Figure 2b). Therefore, the anatoxin-a maximum could not occur during the early exponential phase, as was observed for all other treatments. These results indicate that exponential growth is important for maximum anatoxin-a production, explaining the low toxin quota maximum recorded for MLA_0.1×Fe_.

While iron and copper altered the growth profiles of strain CYN52, there was little change in the trend of anatoxin-a production. The maximum anatoxin-a quota for MLA_2×Fe_, the highest iron concentration that was not growth-inhibiting, occurred on Day 6. This value was significantly higher than all the other iron treatments on the same day. The maximum anatoxin-a quota for MLA_1×Fe_ was significantly higher than all anatoxin-a quota between all iron treatments from Day 22 to 49. The anatoxin-a maxima for MLA_2×Fe_ and MLA_1×Fe_ were statistically significantly higher than the quota for other iron media on adjacent days including MLA_0.1×Fe_ on Day 12 and MLA_2×Fe_ on Day 9 and 12. The maximum anatoxin-a quota recorded for each copper treatment were within the 95% confidence interval of each other. All anatoxin-a quota maxima for copper treatments were significantly higher than the anatoxin-a quota recorded for all treatments from Day 32 to 49 (Figure 3).

**Figure 4:**
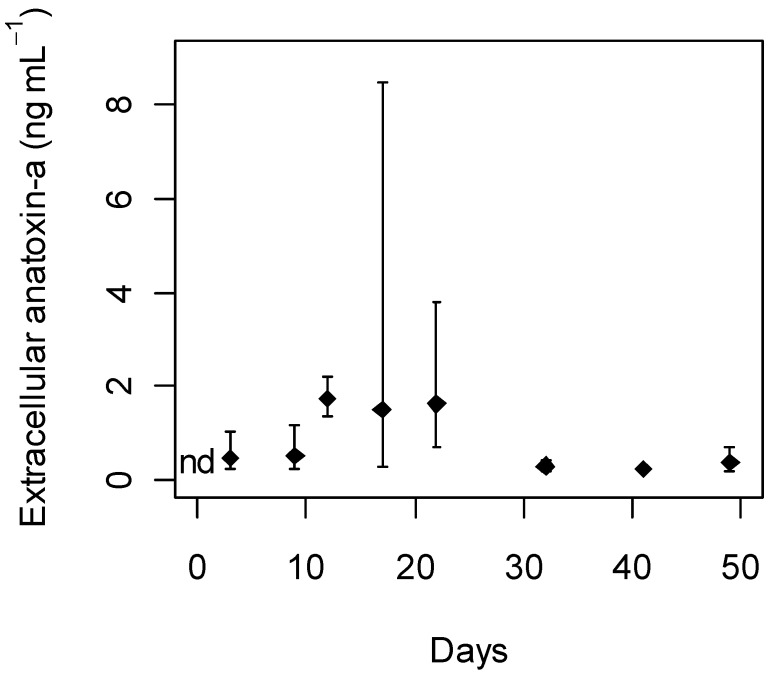
Extracellular anatoxin-a detected in *Phormidium autumnale* (CYN52) growth experiment under standard MLA_1×Fe,1×Cu_. Extracellular anatoxin-a is recorded as mean concentration with 95% confidence intervals (*n* = 3); nd, not detected.

Anatoxin-a was not detected in any of the culture media blanks. Every sampling point of treatment MLA_1×Fe,_ _1×Cu_ was analysed for extracellular anatoxin-a. The majority of anatoxin-a was found to be intracellular. Extracellular anatoxin-a was initially detected on Day 3 and was also identified from Day 9 until the end of the experiment (Figure 4).

The observed variation in extracellular anatoxin-a concentrations (Figure 4) is not likely to be influenced by iron and copper concentration. Stevens and Krieger [56] observed that photolytic degradation of anatoxin-a occurred on a faster time-scale compared to non-photolytic degradation catalysed by iron or copper. The photolytic degradation occurred within a couple of hours. The non-photolytic degradation occurred over a couple of weeks.

**Figure 5:**
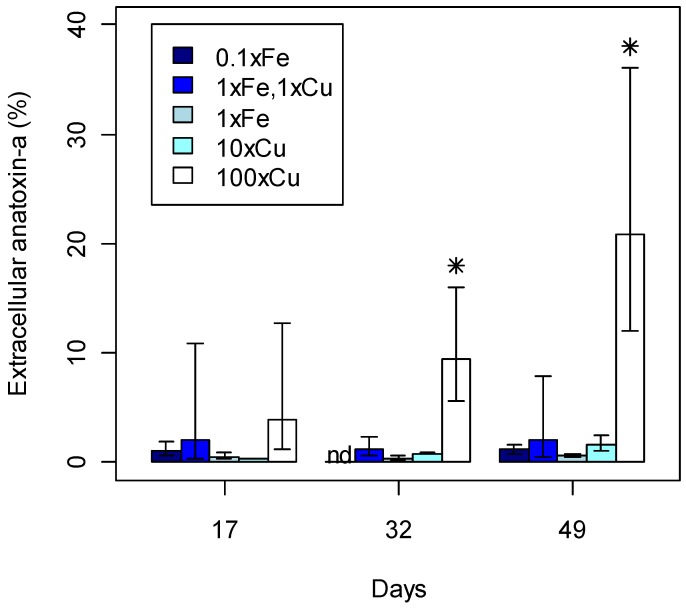
Percentage of extracellular anatoxin-a from total intra- and extracellular anatoxin-a detected for each treatment (Fe = iron, Cu = copper) during the late exponential and stationary phase. 95% confidence intervals are included; 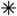 statistically significant concentrations per day; nd, not detected.

Anatoxin-a is expected to be mostly intracellular under beneficial growth conditions [6]. The highest extracellular anatoxin-a concentrations detected in MLA_1×Fe,1×Cu_ was 2.5 ± 1.9 ng mL^−1^ recorded on Day 17 (Figure 4). Therefore, the modified MLA treatments were only analysed in the mid-exponential and stationary phase on Day 17, 32, and 49 (Figure 5). Under high copper concentrations (250 μg L^−1^ Cu), a significantly higher percentage of extracellular anatoxin-a to total anatoxin-a was detected on Day 32 and 49 compared with other treatments. These high anatoxin-a concentrations could be due to cell lysis [6] or deformation of the cell wall by copper [57] allowing anatoxin-a to leak into the surrounding culture medium. Dead trichomes with deformed cell walls were observed on Day 49 for the high copper treatment, supporting the suggestion that anatoxin-a was released through cell lysis. Mass cell-lysis events in other toxic cyanobacteria have caused human poisonings resulting in hospitalisation [58,59] and death [60].

The experimental design used in this study was adapted from Esson *et al.* [61] to incorporate cyanotoxin analysis of benthic cyanobacteria throughout the growth cycle. The development of their method during this work will allow experiments to be undertaken to enhance understanding of the influences of a range of parameters on growth and toxin production in benthic cyanobacteria. Sivonen [6] investigated gravimetric microcystin production in *Oscillatoria agardhii* using a similar approach. However, in the *O. agardhii* study 3–6 samples were pooled for each data point. In the current *P.* *autumnale* study three replicates were used for each data point, allowing for statistical analyses to be carried out. The use of cell concentration rather than biomass allows for a better estimation of growth.

### 2.4. Biological Role of Anatoxin-a

Assuming that anatoxin-a is a secondary metabolite, when an organism is under stress and struggling to grow, toxin production may be up or down regulated. In this study this was observed under very high iron and copper, or very low iron conditions. The physiological and ecological role of anatoxin-a has not been identified [27,46]. The maximum toxin quota occurred early in the growth cycle for all cultures, usually corresponding with the spread of trichomes over all available substrata. This observation suggests that anatoxin-a may be involved in initial colonisation, although confounding this hypothesis is the occurrence of anatoxin-a in planktonic species where attachment is not required [6]. Anatoxin-a may be involved with intra- or inter-species interactions, including allelopathic or chemotaxic signalling between other *P. autumnale* cells or bacteria, during this initial growth phase. Similar suggestions of the involvement of microcystin in cell-cell interactions and colony formation have been made for *Microcystis* [62,63]. It has been suggested that the biological role of cyanotoxins could be as a defence mechanism [10]. The green alga *Chlamydomonas reinhardtii* is paralysed by exposure to anatoxin-a [64]. However, anatoxin-a is produced in unialgal axenic culture, suggesting that this toxin is not produced as a response to other organisms, even if it may on occasions adversely affect some algal species. 

## 3. Methods

### 3.1. Experimental Design and Harvest

*Phormidium autumnale* strain CYN52 was obtained from the Cawthron Institute Culture Collection of Micro-algae (CICCM, Nelson, New Zealand; http://cultures.cawthron.org.nz/). This strain is known to produce anatoxin-a but not homoanatoxin-a [8]. Strain CYN52 was grown in batch culture in 500 mL MLA culture medium [65] to obtain sufficient wet weight inoculum for the experiment.

Metal concentrations were chosen to span concentrations found in freshwaters as well as concentrations previously reported to be toxic to cyanobacteria. MLA and five variations of this culture medium were prepared in 5 L acid-washed plastic carboys by modifying either the FeCl_3_·6H_2_O or CuSO_4_·5H_2_O concentrations (Supporting Information Table S3). These salts are the same as those used to prepare standard MLA medium [65]. Nominal concentrations in standard MLA, without modifications, are 400 μg L^−1^ Fe and 2.5 μg L^−1^ Cu and designated MLA_1×Fe,1×Cu_. Copper sulfate concentrations were increased to 25 and 250 μg L^−1^ Cu in MLA_10×Cu_ and MLA_100×Cu_ respectively. Iron chloride concentrations were decreased to 40 μg L^−1^ Fe in MLA_0.1×Fe_, or increased to 800 μg L^−1^ in MLA_2×Fe_, and 4000 μg L^−1^ Fe in MLA_10×Fe_. The concentration of sulfate increased from 27.8 to 28.2 mg L^−1^ when copper was increased to 100× the standard MLA copper concentration. The concentration of chloride increased from 7.18 to 9.74 mg L^−1^ when iron was increased from 0.1× to 10× standard MLA iron concentration. The minimal change in the counter ion concentration (sulfate and chloride) was assumed to have a negligible overall effect on the cells from compared with that the effects caused by changes in iron or copper concentrations.

The experimental design was modified from Esson *et al.* [61]. Aliquots (30 mL) of culture media were pipetted into pre-numbered, pre-weighed, sterile polystyrene culture containers (70 mL, Labserv). Each container was inoculated with 7 mg (±0.5 mg) wet weight of CYN52. Five MLA control samples in duplicate, consisting of culture medium (30 mL) containing no inoculum, were set up for each treatment. Cultures and controls were incubated at 18 °C (± 1 °C), under 36 µE m^−2^ s^−1^ of light (12 h light-dark cycle). To minimise variation in growth due to different light intensities reaching each culture container, the container positions were randomised at each harvest date according to a random number generator ([66]www.random.org/sequences). To adjust for any changes in light intensity due to harvest of cultures, gaps from harvested containers were replaced with culture containers containing 30 mL of water. 

Two sets of triplicate cultures from each treatment were harvested on ten sampling dates spaced between three and ten days apart over 49 days (Supplementary Information Table S4); one set was for growth analysis and the other for anatoxin-a analysis. An additional two sampling dates were included for the standard treatment (MLA_1×Fe,1×Cu_) to gain extra data for the early stages of the growth profile. Duplicate MLA control samples were harvested with every second or third sampling date for each treatment. These control samples were used to test changes in medium composition due to non-biological processes.

### 3.2. Growth Analysis

At each sampling date, one set of triplicate samples was preserved in Lugol’s iodine and stored in the dark until analysis for cell enumeration. When ready for analysis, the samples were homogenised (2–5 min) by Ultra-Turrax (IKA Laborteknik, Germany). Homogenisation broke up the mat and fragmented trichomes into more uniform lengths. The Ultra-Turrax probe was rinsed twice with Milli-Q water (5 mL), and the rinsates added to the sample. A subsample of 0.25–5 mL, depending on the concentration of cells, was transferred to an Utermöhl chamber [67] and left to settle in the dark (2–24 h).

All trichomes in the Utermöhl chamber were measured along one transect at 400× magnification on an inverted microscope (Olympus CK40 or ITM). If the number of trichomes counted was less than 60, two or more transects were assessed to ensure that approximately 100 trichomes were measured for each sample. Cell lengths (n > 50), from six of the original homogenised cultures (Day 0, 3, 9, 17, 32 and 49) for each treatment, were measured at 800–1000× magnification using an Olympus light microscope (BX51). The average cell length for each treatment was calculated and used to determine the number of cells per trichome measured in the Utermöhl chamber. These data were used to calculate the number of cells in the original 30 mL culture.

### 3.3. Culture Medium Analysis

From each of the second triplicate sample set and control samples, a subsample (10 mL) of culture medium was removed. This subsample was acidified (8M HNO_3_, 500 µL) and ICP-MS (Agilent 7500 cx) was used to determine iron and copper concentrations in culture media**.** Sub-samples (10 mL) were stored in acid-washed polypropylene test-tubes and acidified with HNO_3_ (8 M, 500 µL). The acidified sub-samples from MLA_2×Fe_ were diluted 4-fold with 2% HNO_3_, prior to analysis. Duplicates and spiked samples (20 µL^‑1^ iron and copper) were analysed after 10 and 20 samples respectively. A six point calibration (0.1–1000 μg L^‑1^) was used. Check standards (0, 2, 20 µg L^‑1^) were analysed after every set of 20 samples. The mean percentage difference (*n* = 26) for duplicates was 6.5% for Cu and 3.6% for Fe. Mean spike recoveries (*n* = 4) were 89.5% and 82.4% for Cu and Fe respectively. Metal results were not recovery corrected. The pH of the remaining medium was measured (SevenEasy meter, Mettler Toledo). Subsamples (1 mL) were collected for extracellular anatoxin-a and nutrient (10 mL) analyses. These subsamples were filtered (GF/C, Whatman) and stored frozen (20 °C) until analysis.

The nutrient subsamples were analysed for phosphate (PO_4_-P), nitrate and nitrite (NO_3_-N + NO_2_-N) using colorimetric methods (US EPA Methods 353.2, 354.1, and 365.1) on an automated analyser (EasyChem Plus, Systea). Phosphate and nitrate samples from the culture samples and growth medium control samples were diluted 100-fold prior to analysis. Working stock solutions of 10 mg L^‑1^ PO_4_-P, 10 mg L^‑1^ NO_3_-N, and 1 mg L^‑1^ NO_2_-N were prepared daily for instrument calibration. Samples were analysed in duplicate with a Milli-Q blank between every sample. The mean of duplicate samples was reported. Two quality control samples were included in every batch. The mean percentage difference (*n* = 75) between sample duplicates were 9.3% and 1.4% for combined nitrogen from nitrate and nitrite sources and phosphorus respectively.

### 3.4. Anatoxin-a Analysis

The biomass from the second triplicate sample set was used for anatoxin-a analysis. The remaining culture medium was carefully decanted or removed by pipette from mats of CYN52. This biomass was lyophilised (FD-1, EYELA, Tokyo Rikaikai Co. Ltd, Tokyo, Japan), weighed, and stored frozen (−20 °C) until analysis. Aliquots of acidified Milli-Q water (5 mL, 0.1% formic acid) were added to each lyophilised sample, ensuring any biomass attached to the culture container wall was transferred into this volume. The sample was sonicated in an ice bath for 30 min (Kudos ultrasonic cleaner bath, Model 250LHC, at 59 KHz), centrifuged (3000 × g, 5 min), and 1 mL of supernatant was removed for analysis of intracellular anatoxin-a.

Intra- and extracellular samples were analysed for anatoxin-a, homoanatoxin-a, dihydroanatoxin-a and dihydrohomoanatoxin-a based on the LC-MS method of Wood *et al.* [9]. Compounds were separated by liquid chromatography (Waters Acquity UPLC, Waters Corp., MA, USA) on a BEH C18 column (1.7 µm, 1 × 50 mm, Waters Corp., MA, USA) and quantified on a Quattro Premier XE triple quadrupole mass spectrometer (Waters-Micromass, Manchester, UK). Certified anatoxin-a (A.G. Scientific, CA, USA) was serial diluted with 0.1% formic acid for instrument calibration. After calibration, check calibration standards were analysed every 5–8 samples to monitor instrument performance. Samples from MLA controls were analysed in each batch as negative controls. Anatoxin-a concentrations were expressed as quantity of anatoxin-a per cell (anatoxin-a quota) rather than normalised to mass or other metabolites such as chlorophyll-a or proteins, which may vary considerably with growth phase.

### 3.5. Statistical Analysis

Experimental design was balanced as all treatments consisted of three replicates. Two-way analysis of variance (ANOVA) was used to determine if there were significant relationships between metal concentration and either growth or anatoxin-a production. *P-*values < 0.05 were considered statistically significant. To determine where significant differences occurred, 95% confidence intervals were calculated for the expected values for each treatment and day. 

Anatoxin-a measurements were not correlated with one particular cell concentration because the three anatoxin-a replicates were different samples from the three cell concentration replicates. Therefore, the anatoxin-a quota was calculated from the average number of cell numbers for the corresponding treatment and day of each anatoxin-a data point. Raw data from growth and anatoxin-a production were transformed logarithmically prior to analysis to ensure residuals were normally distributed. The validity of this assumption was checked using QQ-plots. Homoscedasticity and misspecification were assessed by plotting residuals against fitted values. Akaike Information Criterion (AIC, [68]) was used to compare statistical models for the same datasets. AIC takes into account both, the likelihood, *i.e.* goodness-of-fit, of the model and the parsimoniousness, *i.e.*, number of parameters, to produce a balanced assessment. Smaller values of AIC correspond to better models with the difference of three usually considered suggestive and difference of seven significant. All statistical analyses were carried out using R version 2.13.1 [69]. 

## 4. Conclusions

Toxin production by benthic cyanobacteria has been largely overlooked, despite the increasing number of reports of these toxin producers and associated fatalities worldwide. In some countries, such as New Zealand, benthic cyanotoxin producers are significantly more problematic than planktonic species. Methods used in this study will significantly advance our understanding of toxin production and growth of benthic cultures. Data from this study, and future experiments using similar protocols, will assist in understanding conditions that favour growth and toxin production. In this study, iron and copper significantly affected growth but not anatoxin-a production. We identified that anatoxin-a production is markedly higher early in the growth cycle. Therefore although biomass is lower during initial colonization and growth, these periods may pose a higher health risk to animal and human users of rivers.

## Supplementary Files

Supplementary File 1 Supplementary Information (PDF, 156 KB)
