# Supplementary material for: Phormidium autumnale Growth and Anatoxin-a Production under Iron and Copper Stress
Source: Toxins (Basel). 2013 Dec 16;5(12):2504–21. doi: 10.3390/toxins5122504 (PMC3873698; doi:10.3390/toxins5122504)
Supplement: Supplementary File 1 — Supplementary Information (PDF, 156 KB) [file toxins-05-02504-s001.pdf]

Supplementary Information

Figure S1. Estimated mean metal concentrations in MLA with 95% confidence intervals measured in *Phormidium autumnale* (CYN52) growth experiments: (**a**) MLA$_{1\times Fe=1\times Cu}$, 400 µg L^{-1} Fe; (**b**) MLA$_{10\times Cu}$, µg L^{-1} Fe; (**c**) MLA$_{100\times Cu}$, 400 µg L^{-1} Fe; (**d**) MLA$_{0.1\times Fe}$, 40 µg L^{-1} Fe; and (**e**) MLA$_{2\times Fe}$, 800 µg L^{-1} Fe; ▲ control, ■ treatment. MLA$_{2\times Fe,1\times Cu}$ corresponds to 800 and 2.5 µg L^{-1} for Fe and Cu treatments, respectively. The reference (MLA$_{1\times Fe,1\times Cu}$) corresponds to 800 and 2.5 µg L^{-1} for Fe and Cu treatments, respectively.

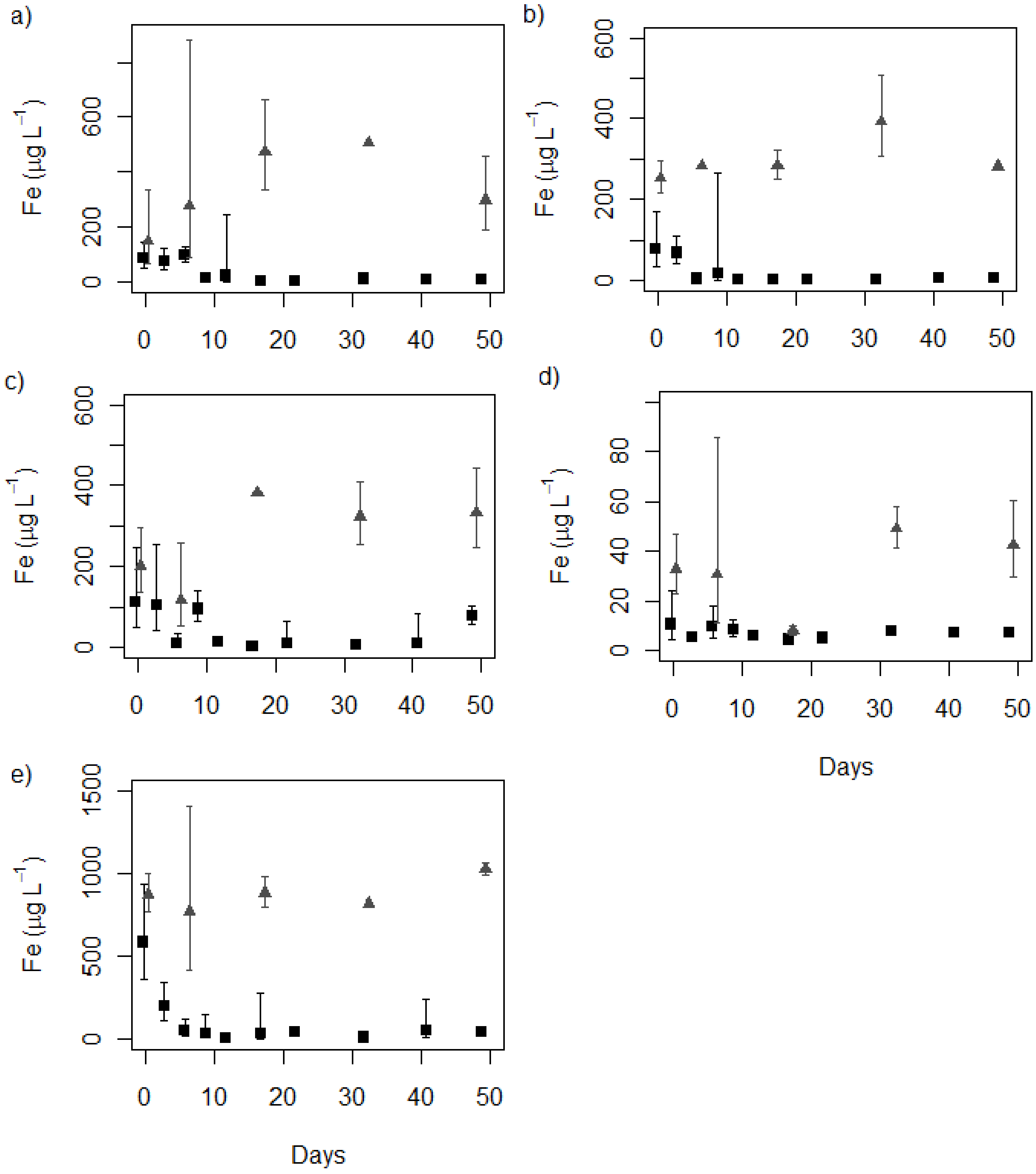

Figure S2. Estimated mean metal concentrations in MLA with 95% confidence intervals measured in *Phormidium autumnale* (CYN52) growth experiments: (**a**) $MLA_{1\times Fe=1\times Cu}$, 2.5 µg L^{-1} Cu; (**b**) $MLA_{10\times Cu}$, 25 µg L^{-1} Cu; (**c**) $MLA_{100\times Cu}$, 250 µg L^{-1} Cu; (**d**) $MLA_{0.1\times Fe}$, 2.5 µg L^{-1} Cu; and (**e**) $MLA_{2\times Fe}$ µg L^{-1} Cu; ▲ control, ■ treatment. $MLA_{2\times Fe,1\times Cu}$ corresponds to 800 and 2.5 µg L^{-1} for Fe and Cu treatments, respectively. The reference ($MLA_{1\times Fe,1\times Cu}$) corresponds to 800 and 2.5 µg L^{-1} for Fe and Cu treatments, respectively.

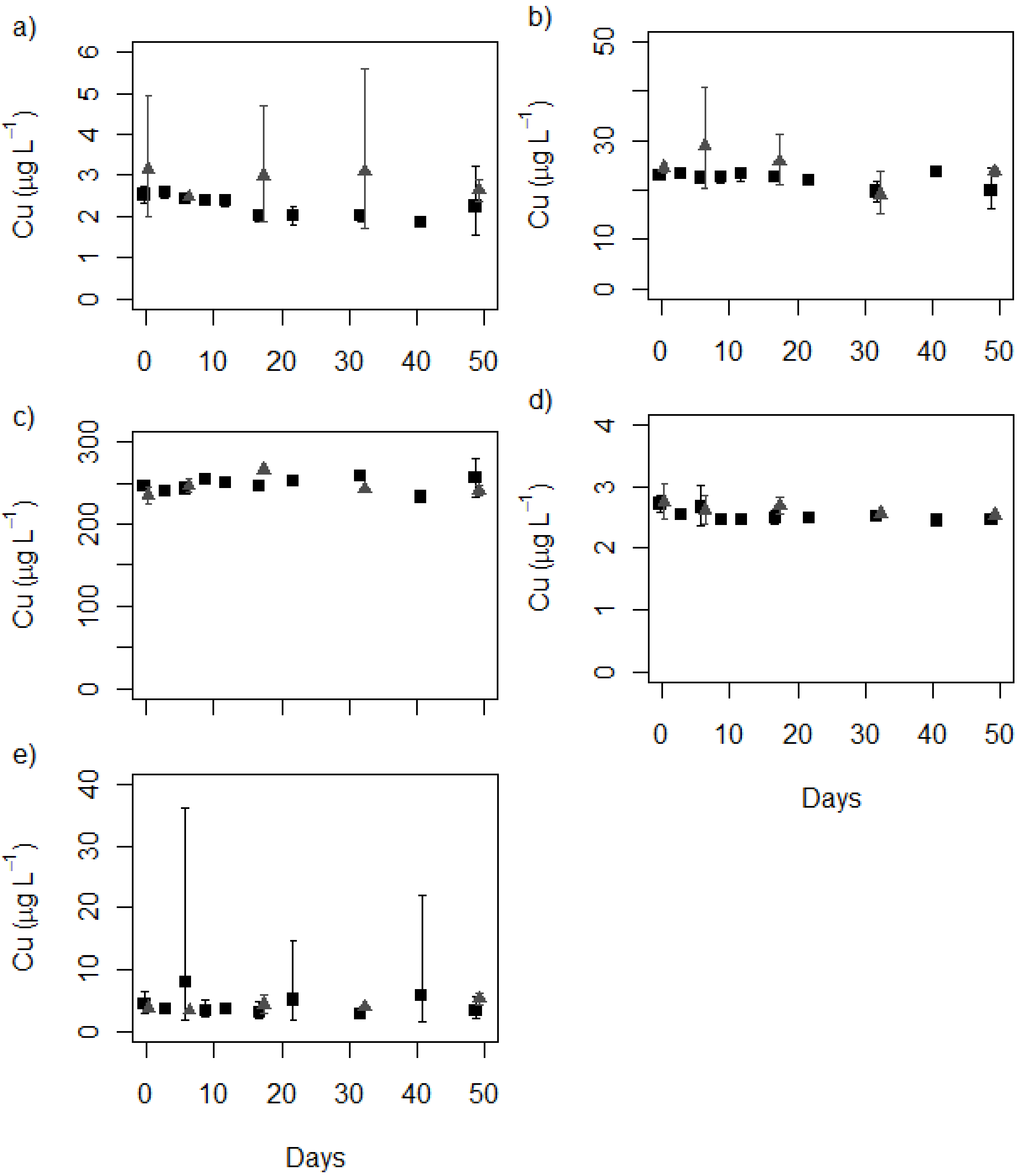

Table S1. Comparison of different growth models using Akaike Information Criteria (AIC) to evaluate the goodness of fit. For each dataset, a linear model (explaining the overall trend of the data over time) was compared to a categorical model (where days were treated as a factor) and intercept model (where time was not a variable, *i.e.*, zero growth assumed). A smaller AIC value indicates a better model.

Growth Model Test for Each Treatment	AIC
**Iron Treatment**	
Categorical model	−41.1
Log-linear model	37.9
Sigmoidal curve model	51.5
**Copper Treatment**	
Categorical model	−1.6
Log-linear model	48.2
Sigmoidal curve model	123

Table S2. Comparison of different anatoxin-a quota models using Akaike Information Criteria (AIC) to evaluate the goodness of fit. For each dataset, a linear model (explaining the overall trend of the data over time) was compared to a categorical model (where days were treated as a factor) and intercept model (where time was not a variable, *i.e.*, zero change in anatoxin-a quota assumed). A smaller AIC value indicates a better model.

Growth Model Test for Each Treatment	AIC
**Copper Treatment**	
Categorical model	48.1
Log-linear model	98. 8
Intercept model	222
**Iron Treatment**	
Categorical model	57.8
Log-linear model	106
Intercept model	137

Table S3. Nominal iron and copper concentrations series for modified MLA culture media used for each treatment in this experiment.

Treatment	Fe [ppb]	Cu [ppb]
$MLA_{1 \times Fe = 1 \times Cu}$*	400	2.5
$MLA_{0.1 \times Fe}$	40	2.5
$MLA_{2 \times Fe}$	800	2.5
$MLA_{10 \times Fe}$	4000	2.5
$MLA_{10 \times Cu}$	400	25
$MLA_{100 \times Cu}$	400	250

Note: * $MLA_{1 \times Fe = 1 \times Cu}$ represents standard MLA medium without modifications.

Table S4. Harvest regime for the iron and copper stressor experiment.*

Harvest day	MLA control samples	Culture samples†	Additional culture samples†
Day 0	✓	✓	
Day 3		✓	
Day 6	✓	✓	
Day 9		✓	
Day 10			✓
Day 12		✓	
Day 13			✓
Day 17	✓	✓	
Day 22		✓	
Day 32	✓	✓	
Day 41		✓	
Day 49	✓	✓	

Notes: *: MLA control samples contain only culture medium of each treatment. Culture samples were inoculated with CYN52 for each treatment. Additional culture samples were inoculated for the standard MLA treatment to collect extra data for the anatoxin-a profile at the early stages of the growth profile; †: Each set of culture samples consists of three cultures for cyanotoxin and three cultures for growth analyses for each treatment.
